# Optimization and Comprehensive Characterization of the Microencapsulation Process for Taro Essence

**DOI:** 10.3390/foods14050754

**Published:** 2025-02-23

**Authors:** Yongxin Song, Yipeng Gu, Aiqing Ren, Xiaochun Li, Shujie Wu, Yuwen Gong, Yanghe Luo

**Affiliations:** 1 School of Chemistry and Pharmaceutical Sciences, Guangxi Normal University, Guilin 541004, China; songyl0805@163.com; 2 Guangxi Key Laboratory of Health Care Food Science and Technology, Institute of Food Science and Technology, Hezhou University, Hezhou 542899, China; renaiqing@126.com (A.R.); 201100019@hzxy.edu.cn (X.L.); sjwu@hzxy.edu.cn (S.W.); 3 College of Biological and Chemical Engineering, Guangxi University of Science and Technology, Liuzhou 545006, China; gongyw1999@163.com

**Keywords:** taro essence, microencapsulation, encapsulation efficiency, physicochemical properties, volatile components

## Abstract

This study investigated the microencapsulation process of natural taro essence and characterized its physicochemical properties. The effects of core-to-wall ratio, T-20/β-CD mass ratio, and ultrasonic time on encapsulation efficiency were systematically investigated. Optimal conditions, identified through orthogonal experiments, included a core-to-wall ratio of 1:10, a T-20/β-CD mass ratio of 1.6:1, and an ultrasonic time of 40 min, resulting in an encapsulation efficiency of 56.10%. The characterization of the microcapsules revealed satisfactory physical properties, including low moisture content, suitable solubility, appropriate bulk density, and good flowability. Particle size distribution analysis showed consistency, and zeta potential measurements indicated stability against agglomeration. Thermal analysis demonstrated enhanced thermal stability, and FT-IR spectroscopy confirmed successful encapsulation through significant interactions between taro essence and β-CD. SEM imaging revealed a heterogeneous morphology, while XRD patterns validated the formation of stable inclusion complexes. An analysis of volatile components indicated the effective encapsulation of key alkanes, with PCA and heatmap clustering analyses confirming the stability of these components during storage. In conclusion, the optimized microencapsulation process significantly enhances the encapsulation efficiency, stability, and thermal properties of natural taro essence microcapsules.

## 1. Introduction

Taro, scientifically known as Colocasia esculenta, is a significant root crop valued for its high starch content, making it a cherished food source [1,2]. The corms of taro are particularly rich in starch and gums, which contribute to its unique properties and enable its use in a variety of culinary applications [3]. Renowned for its elevated water content and the profound aroma it emanates during culinary preparation, taro notably elevates the sensory appeal of food [4]. However, it is important to note that taro’s inherent flavor tends to diminish or even disappear after prolonged storage or processing [5]. This presents a challenge to the marketability of taro-derived products and affects consumer acceptance. Thus, understanding the complex chemical composition of taro is crucial for developing effective processing methods that preserve its flavor and overall quality [3]. To enhance the preservation of taro’s flavor, extracting edible essences from natural plants has been proposed to improve its utilization in food products, ensuring the retention of its rich and distinctive flavor over extended periods [1].

Microencapsulation technology, a pivotal approach for safeguarding volatile and degradable plant flavors, particularly essential oils, involves encapsulating these minute particles or droplets within a protective coating wall composed of either uniform or non-uniform matrices, yielding particles typically spanning 1 to 1000 μm in diameter [6,7]. This technology employs diverse methods, notably including beta-cyclodextrin (β-CD), polymer microcapsules, and inorganic microcapsules, to form a barrier that enhances stability and governs the controlled release of plant essences [8,9]. β-CD stands out as a highly efficacious encapsulating material for plant essential oils, forming robust non-covalent complexes that bolster stability and mitigate the detrimental effects of external stressors like light, heat, and oxygen [10,11]. By meticulously manipulating the structure and properties of the inclusions, the encapsulation process facilitates precise control over fragrance release rates [11]. Furthermore, β-CD has demonstrated its proficiency in encapsulating a broad spectrum of essential oil molecules, enhancing solubility and overcoming volatility constraints, as exemplified by Mosla Chinensis, Rosmarinus officinalis, Lavandula angustifolia, and Citrus aurantium [10,11,12]. Polymeric nanocarriers are also harnessed to encapsulate essential oils derived from diverse plant tissues, safeguarding them from external threats and enabling controlled release, thereby augmenting their bioavailability and efficacy across various applications [13].

Microencapsulation technology not only enables efficient fragrance adsorption and embedding but also ensures uniform dispersion and stable suspension in diverse application systems, contributing to the longevity and effectiveness of plant essences in various products [8,9,14]. However, commercial taro flavorings often depend on synthetic additives for stability and dispersibility, which may not align with clean-label trends or consumer preferences for natural ingredients. The high viscosity and poor dispersibility of taro extracts limit their industrial applications and versatility in food formulations. Additionally, the current research landscape pertaining to the encapsulation of taro essence remains nascent, characterized by a notable scarcity of studies investigating the application of this technology. Therefore, the present study aims to fill this gap by employing the saturated solution methodology to prepare β-CD inclusion complexes with taro essence. Ultrasound-assisted rapid encapsulation was used to generate smaller and more uniform particle sizes, enhance encapsulation efficiency, and promote the formation of stable inclusion complexes through improved interactions between the core and wall materials [15,16]. Subsequent to the preparation, a comprehensive evaluation of the physicochemical properties of the resulting microcapsules was undertaken. This assessment encompassed the utilization of DSC, TGA, SEM, and FT-IR, providing insights into the structural and thermal characteristics of the microcapsules. Furthermore, HS-SPME-GCMS was employed to identify the volatile components and analyze the temporal evolution of the essence composition during storage. In parallel, the potential of utilizing natural taro essence microcapsules for flavor encapsulation has been explored, showcasing their innovative applications in food products.

## 2. Materials and Methods

### 2.1. Materials and Chemicals

Fresh taro was purchased from Taixing Supermarket (Hezhou, China); β-cyclodextrin was purchased from Huaxing Biochemical Co., Ltd. (Mengzhou, China); ethanol and dichloromethane were obtained from Guangdong Guanghua Technology Co., Ltd. (Guangzhou, China); commercial taro essence was purchased from Xinai Food Technology Co., Ltd. (Lianyungang, China); and Tween-20 was sourced from RHAWN Chemical Reagent Co., Ltd., (Shanghai, China). All chemical reagents were of analytical grade.

### 2.2. Extraction of Taro Essence

The fresh taro was washed to remove impurities, wrapped in cotton cloth, and steamed in boiling water for one hour. After peeling and cutting into small pieces, it was placed in a conical flask with dichloromethane at a 1:2 ratio. The flask was sealed and the mixture was extracted for 24 h at room temperature. The solution was then filtered to remove solid impurities, centrifuged at 5000 r/min for 15 min, and the supernatant was concentrated by rotary evaporation to remove dichloromethane. Finally, the extract was freeze-dried to obtain taro essence.

### 2.3. Preparation of Taro Essence Microcapsules

A measured amount of taro essence and the emulsifier Tween-20 (T-20) were then added to the saturated aqueous solution of β-cyclodextrin (β-CD). The mixture was thoroughly agitated to ensure complete mixing of the components. Subsequently, it was subjected to ultrasonic treatment at 150 W for 30 min at 25 °C to enhance the dispersion of taro essence within the solution. Following the ultrasonic treatment, the mixture underwent homogenization at a speed of 12,000 r/min for 15 min to achieve a uniform emulsion. After homogenization, the mixture was stored overnight in a refrigerator to allow for proper settling and stabilization. The next day, the mixture was transferred to a centrifuge tube and centrifuged at 10,000 r/min for 10 min to separate the solid and liquid phases. The precipitate obtained from centrifugation was then freeze-dried for 48 h, resulting in the formation of taro essence/β-CD microcapsules.

### 2.4. Calculation of the Encapsulation Efficiency and Yield of Essence Microcapsules

A 0.1 g sample of essence microcapsules was accurately weighed and transferred to a centrifuge tube, where 5 mL of anhydrous ethanol was added and gently shaken to wash away residual surface essence (since β-CD, the wall material, is insoluble in ethanol). Subsequently, the microcapsules were then collected, and residual surface ethanol was evaporated. The cleaned microcapsules were disrupted by adding 10 mL of deionized water (to dissolve β-CD) and 10 mL of dichloromethane (to extract the encapsulated essence), followed by ultrasonication (40 kHz, 30 min) and centrifugation (10,000 rpm, 10 min, 4 °C). The organic phase was separated, concentrated under reduced pressure, and freeze-dried to determine the mass of encapsulated essence (*m*_1_), while the aqueous phase was freeze-dried to obtain the mass of β-CD (*m*_2_). The encapsulation efficiency (EE), representing the percentage of initial essence (*m*_0_) successfully encapsulated, was calculated as follows:

(1) $$EE(\%)=\frac{m_{1}}{m_{0}}\times 100$$

The microcapsule yield (MY), defined as the percentage of the total mass of encapsulated essence (*M*_1_) relative to the total initial mass of the core material (essence, *M*_0_) and wall material (β-CD, *M*_2_), was calculated as follows:

(2) $$MY\left(\%\right)=\frac{M_{1}}{M_{0}+M_{2}}\times 100$$

### 2.5. Single-Factor Experiments in the Preparation of Microcapsules

In optimizing the preparation process of inclusion complexes, a series of single-factor experiments were conducted to investigate the effects of core-to-wall ratio, emulsifier T-20 to β-CD ratio, and ultrasonication time on the encapsulation efficiency of microcapsules. EE served as the key indicator for optimizing preparation conditions, analyzed using SPSS software (Version 19.0, IBM Corporation, Armonk, NY, USA). Encapsulation efficiency served as the primary metric for identifying optimal preparation conditions. Each experimental condition was tested in triplicate to ensure the consistency and reliability of the results. Initially, the influence of core-to-wall ratio was examined while maintaining a fixed T-20/β-CD mass ratio of 1.2:1 and constant ultrasonication time of 30 min. Various core-to-wall ratios (1:2, 1:4, 1:6, 1:8, 1:10, and 1:12) were evaluated to assess their impact on encapsulation efficiency. Next, the effect of the emulsifier ratio was investigated with a consistent core-to-wall ratio of 1:10 and ultrasonication time of 30 min. Different T-20/β-CD mass ratios (0.8:1, 1:1, 1.2:1, 1.4:1, and 1.6:1) were tested to determine their influence on encapsulation efficiency. Lastly, the impact of ultrasonication time was explored while maintaining a core-to-wall ratio of 1:10 and a fixed T-20/β-CD mass ratio of 1.2:1. Ultrasonication durations of 20 min, 30 min, 40 min, 50 min, and 60 min were examined to assess their effect on encapsulation efficiency.

### 2.6. The Orthogonal Experiment for Microcapsule Preparation

After conducting single-factor experiments, the wall-to-core ratio (A), T-20/β-CD mass ratio (B), and ultrasonic time (C) emerged as pivotal variables influencing the microencapsulation process. These factors significantly impact the encapsulation efficiency of taro flavor essence microcapsules, a critical parameter ensuring their stability and efficacy. To optimize the parameters, a 3-factor, 3-level orthogonal experimental design was implemented using Design-Expert software (Version 13.0, Stat-Ease Inc., Minneapolis, MN, USA), with factors A, B, and C and their respective levels defined in Table 1. This systematic approach explores parameter combinations to maximize encapsulation efficiency while evaluating interactions and synergies, establishing a robust framework for taro flavor essence microencapsulation.

### 2.7. Determination of Moisture Content

The moisture content was determined using the gravimetric method [4]. Briefly, 1.0 g of microcapsule powder was accurately weighed and heated at 105 °C for 3–5 h. After cooling to room temperature in a desiccator, the sample was reweighed. This process was repeated until a constant weight was achieved, and the final data were recorded.

(3) $$Moisture\;content\;\left(\%\right)=\left(1-\frac{m_{1}}{m_{0}}\right)\times 100$$

Here, *m*_0_ represents the weight of the untreated microcapsules, and m_1_ denotes the weight of the microcapsules after treatment.

### 2.8. Measurement of Solubility

The solubility of the microcapsule powder was evaluated following the methodology outlined by Santos et al. [17]. Initially, 1 g of the microcapsule powder was precisely weighed and transferred into a 25 mL beaker. Subsequently, 15 mL of distilled water was added to the beaker, and the mixture was agitated at a speed of 1000 r/min for 5 min at room temperature to ensure complete dissolution of the powder. The resulting solution was then transferred into a centrifuge tube and centrifuged at 6000 r/min for 10 min. The supernatant was discarded, and an additional 15 mL of distilled water was added to the precipitate at room temperature. The precipitate was thoroughly dissolved by stirring, followed by another centrifugation step at 6000 r/min. The supernatant was discarded again, and the remaining precipitate was transferred into a pre-weighed beaker (*M*_1_). The beaker containing the precipitate was then dried in an oven at 105 °C until a constant mass (*M*_2_) was achieved. Finally, the solubility of the microcapsule powder was calculated using Formula (4) expressed as a percentage.

(4) $$Solubility\left(\%\right)=\left(1-\frac{M_{2}-M_{1}}{\left(1-\omega \right)\times M}\right)\times 100$$

In the formula, *M*_1_ represents the mass of the beaker in grams; *M*_2_ represents the mass of the beaker with the precipitate in grams; *M* represents the mass of the sample in grams; and *ω* represents the water content of the sample in percentage.

### 2.9. Measurement of the Angle of Repose

A standardized measurement procedure was implemented to precisely ascertain the angle of repose for the taro essence microcapsules. A 1 g sample of the microcapsules was carefully poured through a vertical funnel onto a flat, level tray, forming a conical pile of powder. This process was repeated three times. After each pour, the height (*H*) of the powder accumulation was measured with a calibrated ruler and recorded in centimeters (cm). Similarly, the radius (*R*) of the base of the conical pile was measured from the center to the outer edge and recorded in centimeters (cm). The angle of repose was then calculated using Formula (5).

(5) $$Angle\;of\;repose=arctan\frac{H}{R}$$

### 2.10. Measurement of the Bulk Density and Tapped Density

A precise mass of 1 g of taro flavor microcapsule powder (*M*) was weighed. The powder was then carefully poured through a small funnel made from folded weighing paper into a 10 mL graduated cylinder, which was kept vertical to prevent uneven accumulation. Once the powder was fully transferred, the cylinder was gently shaken to ensure uniform packing. The volume of the powder (*V*_a_) was recorded, representing the volume of the taro flavor essence microcapsules. This process was repeated three times, and the bulk density (*ρ*_a_) was calculated using Formula (6).

(6) $$Bulk\;density\;(\rho_{a})=\frac{M}{V_{a}}$$

Subsequently, the taro flavor essence microcapsules were gently vibrated to achieve a uniform distribution and compacted until no further settling was observed, ensuring a level packing surface. The packed volume of the sample in the cylinder was recorded (*V*_b_), and this process was repeated three times. The tapped density (*ρ*_b_) was then calculated using Formula (7) based on these measured packed volumes.

(7) $$Tapped\;density\left(\rho_{b}\right)=\frac{M}{V_{b}}$$

### 2.11. Measurement of the Flowability and Cohesiveness

In assessing the flow and cohesion properties of taro essence microcapsules, the Carr’s Index (CI) and Hausner Ratio (HR) have become widely recognized as industry-standard measurement techniques [18,19]. These metrics proficiently quantify the packing behavior of microcapsule particles in both static and dynamic environments, thus providing insights into their flowability and cohesiveness attributes. Firstly, the Carr’s Index (CI) is calculated by comparing the bulk density (*ρ*_a_) of the microcapsule powder to its tapped density (*ρ*_b_). The formula is given as follows:

(8) $$CI=\frac{\rho_{a}-\rho_{b}}{\rho_{a}}\times 100$$

Subsequently, the Hausner Ratio (HR) is calculated as the ratio between the bulk density (*ρ*_a_) and the tapped density (*ρ*_b_). Its mathematical formulation is as follows:

(9) $$HR=\frac{\rho_{a}}{\rho_{b}}$$

### 2.12. Measurement of the Particle Size and Zeta Potential

Solutions of β-CD, taro essence microcapsules, and blank microcapsules were prepared at a standardized concentration of 0.5 mg/mL using ultrapure water. These solutions were then carefully dispensed into designated sample cells to avoid cross-contamination. The particle size and zeta potential of each sample were measured at a controlled temperature of 25 °C using the Malvern Zetasizer Nano ZS90 (Malvern, UK). A rigorous procedure was followed involving the execution of three independent scans (replicates) for each sample to guarantee the accuracy and reproducibility of the obtained results.

### 2.13. Measurement of the Scanning Electron Microscopy (SEM)

Prior to Scanning Electron Microscopy (SEM) analysis on a JSM-5600LV SEM instrument (JEOL, Tokyo, Japan), the β-CD, taro essence microcapsules, and blank microcapsules were prepared by undergoing a gold sputter coating process for 30 s using an ion sputter coater (Zhengzhou Saike Instrument Co., Ltd., Zhengzhou, Henan, China). Subsequently, the samples were examined at an accelerating voltage of 2 kV under various magnifications. This method facilitated a comprehensive examination and detailed capture of their morphological characteristics, allowing for an in-depth analysis of surface features and structural differences among the samples.

### 2.14. Measurement of the Fourier Transform Infrared (FT-IR) Spectrum and X-Ray Diffraction (XRD) Patterns

The Fourier Transform Infrared (FT-IR) spectra were acquired utilizing a PerkinElmer spectrophotometer (Los Angeles, CA, USA). Samples comprising 2 mg each of β-CD, blank microcapsule, taro essence microcapsules, and taro essence were prepared. Each sample was thoroughly mixed with 200 mg of potassium bromide (KBr), subsequently pressed into a pellet, and positioned within the sample holder. The FT-IR spectra were then scanned within the wavenumber range of 4000–400 cm^−1^, at a resolution of 4 cm^−1^. The quality of the prepared taro essence microcapsules and the blank microcapsule were assessed via X-ray Diffraction (XRD) analysis. The XRD patterns of the samples were recorded over a broad range of Bragg angles (2θ) from 5° to 90°, at a scanning rate of 2°/min, utilizing an Ultima IV instrument (Tokyo, Japan). This instrument was operated at 40 kV and 30 mA, employing metal Cu kα radiation with a wavelength of 1.5405 A.

### 2.15. Measurement of the Thermogravimetric Analysis (TGA) and Differential Scanning Calorimetry (DSC)

The thermal stability of various samples, including β-CD, blank microcapsules, taro essence microcapsules, and taro essence, were exhaustively examined through thermogravimetric analysis (TGA) utilizing a TGA Q500 instrument (New Castle, DE, USA). In this procedure, each sample weighing approximately 10 mg was subjected to dynamic measurements spanning from 20 °C to 600 °C, at a controlled heating rate of 10 °C/min. To mitigate thermal oxidation, a constant nitrogen flow of 60 mL/min was maintained throughout the analysis. Furthermore, the thermal properties of these samples were thoroughly assessed by differential scanning calorimetry (DSC) employing a Discovery DSC 25 auto system (New Castle, DE, USA). The DSC scans were conducted over a temperature range of 30 °C to 250 °C, at a scan rate of 10 °C/min, utilizing 5 mg samples under a nitrogen atmosphere maintained at 50 mL/min. The heating curves generated from these DSC runs were meticulously analyzed to elucidate the influence of the filler, acting as a nucleating agent, on the thermal behavior of the processed samples.

### 2.16. Analysis of Volatile Compounds by HS–SPME–GCMS

Headspace-Solid-Phase Microextraction-Gas Chromatography–Mass Spectrometry (HS-SPME-GC-MS) was employed to determine the volatile profile of microcapsules during a 10-day storage period at room temperature. Samples were enclosed in 20 mL headspace vials, fortified with an appropriate quantity of 2,4,6-trimethylpyridine (TMP) as the internal standard. The vials were conditioned at 80 °C for 10 min prior to extraction, which was conducted for 50 min using a pre-conditioned 50/30 μm DVB/CAR/PDMS gray fiber at 270 °C for 30 min. GC separation was achieved on a TG-5MS capillary column (30 m × 0.25 mm × 0.25 μm) with helium as the carrier gas at 1.5 mL/min. A splitless injection technique was adopted, and the oven temperature program was initiated at 45 °C (held for 3 min), ramped to 90 °C at 3 °C/min (held for 2 min), then to 150 °C at 2 °C/min (held for 3 min), and finally to 250 °C at 8 °C/min (held for 3 min). The inlet temperature was maintained at 250 °C. MS detection employed electron impact ionization (EI) with a scan range of 30–500 *m/z*, an electron energy of 70 eV, an ion source temperature of 230 °C, a transmission line temperature of 250 °C, and full scan monitoring. Compounds were qualitatively assigned through manual interpretation and NIST 2014 library matching, with a threshold of either forward and reverse match scores exceeding 800 or a single match score above 900 (on a 1000-point scale). Quantitative determination of each volatile compound relied on TMP as the internal standard, according to the following formula:

(10) $$D=\frac{V_{1}\times 0.2}{V_{2}\times m}$$

Here, D is the concentration of the volatile compound (µg/g), *V*_1_ is the peak area of the volatile compound, *V*_2_ is the peak area of the internal standard, 0.2 is the amount of the internal standard (µg), and *m* is the mass of the sample (g).

### 2.17. Statistical Analysis

The data were presented in the form of mean ± standard deviation and subjected to statistical analysis utilizing IBM SPSS Statistics software. To ascertain the statistical significance of the observed variations, a One-way Analysis of Variance (ANOVA) was conducted. The level of significance among the compared samples was established at *p* < 0.05, indicating a 95% confidence interval.

## 3. Results and Discussion

### 3.1. Effects of Core-to-Wall Ratio, T-20/β-CD Mass Ratio, and Ultrasonic Time on Microcapsule Embedding Efficiency

**Figure 1:**
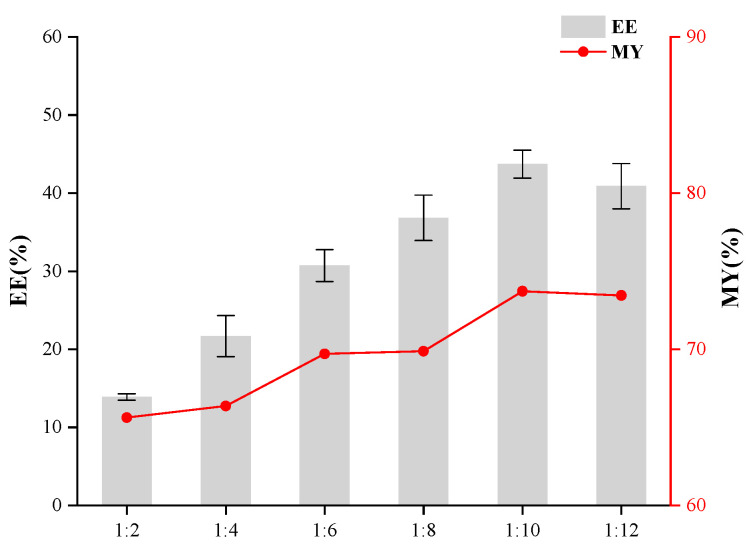
Effect of the mass ratio of taro essence and β-CD on microcapsule embedding efficiency.

**Figure 2:**
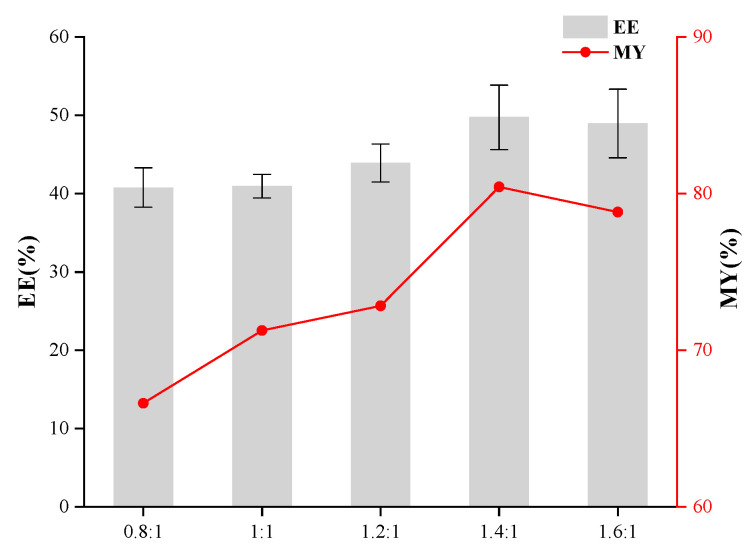
Effect of the mass ratio of T-20 and β-CD on the embedding effect of microcapsules.

**Figure 3:**
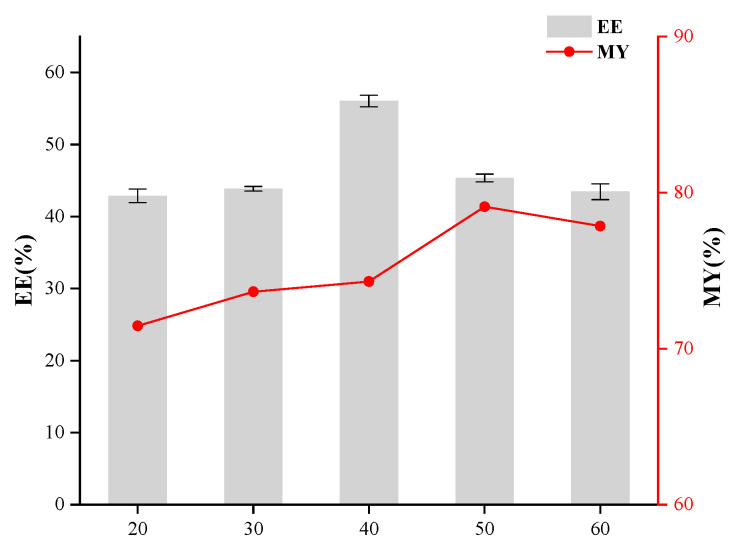
Effect of ultrasonic time on the embedding effect of microcapsules.

The individual effects of the core-to-wall ratio, the T-20/β-CD mass ratio, and ultrasonic time were evaluated to optimize the preparation process of inclusion complexes and determine their respective impacts on enhancing the encapsulation efficiency of the resulting microcapsules. As depicted in Figure 1, the use of taro essence as the core and β-CD as the wall material was examined, with a focus on elucidating the impact of their mass ratio on the encapsulation efficiency of the synthesized microcapsules. Encapsulation efficiency (EE) and microencapsulation yield (MY) initially increased and then decreased with the elevation of the core-to-wall ratio. An optimal EE of 43.70% was achieved at a core-to-wall ratio of 1:10. Beyond this point, further increasing the wall material content led to a decline in encapsulation efficiency. Thus, a core-to-wall ratio of 1:10 was identified as optimal for maximizing microcapsule performance and efficiency. As shown in Figure 2, the influence of the T-20/β-CD mass ratio on the encapsulation performance of microcapsules exhibited a trend where both EE and MY initially increased and then decreased with the addition of T-20. The maximum EE of 49.69% was achieved at a ratio of 1.4:1. When the T-20 content was too low, the membrane became excessively thin, leading to easy rupture during agitation, which compromised the microcapsule morphology and reduced the encapsulation efficiency. As a surfactant, an appropriate increase in T-20 volume fraction aids in minimizing interfacial tension between phases, facilitating efficient encapsulation [20,21]. Hence, the optimal mass ratio of T-20 to β-CD was determined to be 1.4:1. As depicted in Figure 3, the encapsulation efficiency and yield initially increased and subsequently declined with the elongation of ultrasonic treatment time, peaking at 40 min with a maximum EE of 56.04%, followed by a decrease. This phenomenon could be attributed to the excessive heat generated during prolonged ultrasonication, accelerating the volatilization of certain compounds within the core material, thereby reducing the encapsulation efficiency [22]. Consequently, the optimal ultrasonic treatment duration for encapsulation was determined to be 40 min.

### 3.2. Orthogonal Experimental Analysis

The orthogonal approach facilitated a systematic examination of how different factor combinations impacted the embedding efficiency, ultimately leading to the identification of optimal conditions that maximized this parameter [23,24]. Building upon the insights gained from single-factor experiments, an orthogonal L9(3^3^) experimental design was implemented to further refine the embedding efficiency. This design considered the core-to-wall ratio (A), T-20/β-CD mass ratio (B), and ultrasonic time (C) as independent variables, with their respective levels detailed in Table 1. The primary metric for evaluating the experimental outcomes was the embedding efficiency. Table 2 presents the results of the orthogonal experiments along with their subsequent analysis, revealing the order of significance for the factors influencing microencapsulation efficiency to be A (core-to-wall ratio) > C (ultrasonic time) > B (the T-20/β-CD mass ratio). The optimal preparation condition was determined to be A_2_B_3_C_2_, specifying a core-to-wall ratio of 1:10, a T-20 to β-CD mass ratio of 1.6:1, and an ultrasonic time of 40 min. Under these optimized conditions, the preparation of taro flavor essence microcapsules yielded an embedding efficiency of 56.10%, which surpassed the efficiencies recorded under all experimental conditions within the orthogonal test table. This substantial improvement underscores the efficacy of the refined formulation and processing parameters in enhancing the encapsulation performance of the taro essence microcapsules.

### 3.3. Analysis of Basic Physical Properties of Taro Essence Microcapsules

**Figure 4:**
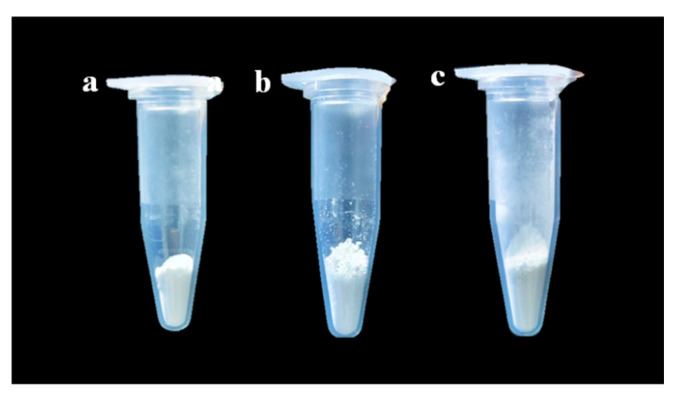
Visual appearance of β-CD (**a**), blank microcapsules (**b**), and taro essence microcapsules (**c**).

Microcapsules exhibit diverse shapes and appearances, typically in the form of fine particles, enhancing their applicability across various fields [7,25]. As shown in Figure 4, β-CD, blank microcapsules, and taro essence microcapsules all appeared as powdery particles. Specifically, both β-CD and empty microcapsules were white, whereas taro essence microcapsules displayed a pale-yellow hue. This coloration change is attributed to the encapsulation of taro essence, which naturally has a yellowish tint, altering the microcapsules’ overall color. The encapsulation of colored or naturally pigmented active ingredients often results in noticeable color shifts in microcapsules, as reported in various studies [26,27,28]. The use of β-CD as a wall material in microencapsulation is significant due to its ability to form inclusion complexes that retain the properties, including color and aroma, of the encapsulated essence [26,29].

Microencapsulation plays a crucial role in maintaining the stability of active ingredients in various food products [14]. The moisture content of microcapsules is a critical quality parameter that directly impacts their stability and shelf life [30]. As shown in Table 3, the moisture content of the taro essence microcapsule powder was 4.76 ± 0.04%. Proper control of moisture content, ideally between 1% and 5%, is essential to preserve the structural integrity and functionality of microcapsules [31]. Maintaining an appropriate moisture level optimizes the design and performance of microcapsules, enhancing their longevity and effectiveness in various applications [30,32].

The solubility of microcapsules significantly affects their performance, influencing the dispersion and release of encapsulated components. Microencapsulation involves enveloping one substance within another, with factors such as polymer solubility, solvent characteristics, and encapsulation efficiency impacting solubility [27]. The results indicated that the solubility of taro essence microcapsules was 61.50 ± 0.05% (Table 3). Fluidity, a crucial indicator for microcapsule products, can be effectively measured using the angle of repose. The angle of repose for taro essence microencapsulated products was determined to be 40.49 ± 0.34°. An angle of repose less than 30° indicates excellent flow characteristics of the powder, while higher values suggest high cohesion or internal friction of the particles [33].

Bulk density (*ρ*_a_) refers to the amount of material contained within a specific volume; higher density values indicate smaller inter-powder pores, minimal air spaces, and reduced oxidative degradation of the encapsulated essence during storage. Packed density (*ρ*_b_) denotes the maximum quantity of microcapsule powder that can be accommodated within a given container [34]. As shown in Table 3, the *ρ*_a_ of the taro essence microcapsules was 0.42 g/mL, and the *ρ*_b_ was 0.48 g/mL. The Carr Index (CI) quantifies powder compressibility and flowability, while the Hausner Ratio (HR) assesses powder cohesiveness. Higher values of both CI and HR suggest poorer flowability and increased stickiness [35,36]. In this experiment, the microcapsules exhibited a CI of 12.72% and an HR of 0.88. Values below 20% for CI and under 1.25 for HR suggest low cohesiveness and excellent flowability [37,38]. The combination of these physical properties, particularly the moderate *ρ*_a_ and *ρ*_b_ along with favorable CI and HR values, underscores the microcapsules’ suitability for applications requiring good dispersion and minimal aggregation during handling and storage. The minimized air pockets within the powder matrix further contribute to preserving encapsulated actives, such as essential oils, by mitigating oxidative degradation, thereby enhancing overall stability and shelf life [39].

### 3.4. Analysis of Particle Size and Zeta Potential of Taro Essence Microcapsules

**Figure 5:**
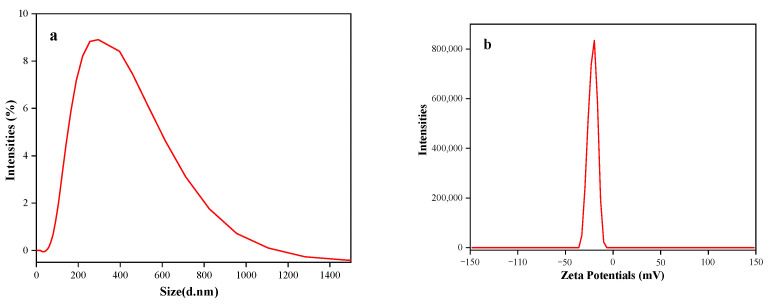
Particle size (**a**) and zeta potential (**b**) of taro essence microcapsules.

The particle size and zeta potential of microcapsules serve as pivotal indicators of their physical attributes, encompassing dimensions and electrical charge characteristics, and they offer insights into their potential for interactions with the ambient environments and biological systems [40,41]. Typically, microcapsules exhibit a diameter spectrum spanning from 1 to 1000 µm, with particles falling below this threshold classified as nanocapsules [6,42]. The analysis presented in Figure 5 showcases a relatively consistent distribution of particle size and zeta potential for taro flavor essence microcapsules. Specifically, the particles ranged in size from 105 to 712 nm, with an average diameter of 352 nm. The zeta potential was ascertained to be −20.8 mV. The particle size exerts a pronounced influence on numerous properties of microcapsules, notably including specific surface area, solubility, and diffusivity [43,44]. Smaller particles contribute to reduced agglomeration, a phenomenon that enhances the stability of microcapsules during both storage and application [45]. Additionally, the zeta potential serves as an indicator of the stability of microcapsules in solution, with large absolute values indicating greater stability due to enhanced electrostatic repulsion, which mitigates particle agglomeration or flocculation [46].

### 3.5. TGA Analysis of Taro Essence Microcapsules

**Figure 6:**
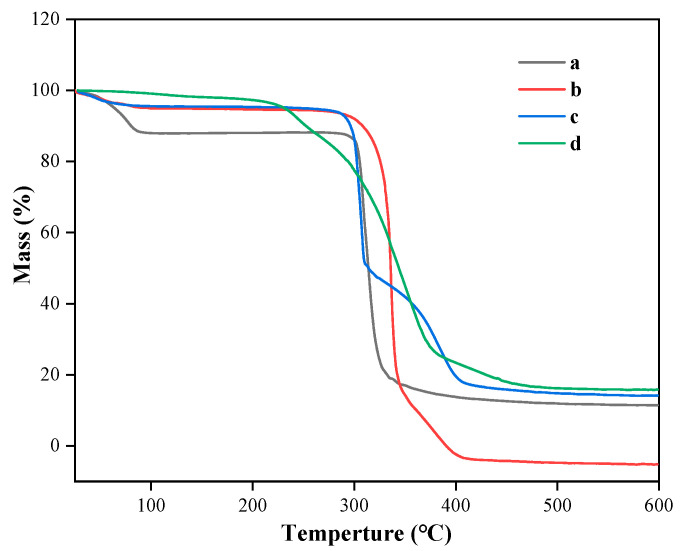
The TGA curves of β-CD (**a**), blank microcapsules (**b**), taro essence microcapsules, (**c**) and taro essence (**d**).

TGA is routinely employed to evaluate the thermal properties of materials and can provide evidence for the formation of inclusion complexes through these thermal behaviors [47]. As depicted in Figure 6, all samples exhibited weight loss in their TG curves. For β-CD, the first stage of weight loss, accounting for 11.7%, commenced at 28 °C and extended to 88 °C, attributed to the evaporation of bound water within and on the surface of β-CD. Subsequently, between 296 °C and 347 °C, a significant weight loss of 69.8% was observed, likely due to the degradation of β-CD [48]. The blank microcapsules exhibited a minor weight loss of 4% starting from 28 °C, likely due to trace moisture in the powder. From 90 °C to 298 °C, the curve remained flat, indicating no further weight loss. Beyond 298 °C, the β-cyclodextrin molecules underwent rapid decomposition, followed by a gradual process until complete decomposition at 410 °C. The taro essence exhibited a gradual weight loss trend starting from 30 °C, with a rapid weight loss of 69.08% occurring between 229 °C and 380 °C, attributable to the high volatility of its components. Between 380 °C and 461 °C, the majority of volatile components were expelled, with an additional loss of 8.86% due to the evaporation of high-boiling-point compounds with larger molecular weights. Beyond 461 °C, the weight of the taro essence remained stable until 600 °C. Compared with β-CD, the microcapsules containing taro essence exhibited a more gradual weight loss profile. An initial weight loss of 5% was observed, followed by a rapid 42.1% weight loss between approximately 280 °C and 309 °C, attributed to the evaporation of taro essence molecules replacing water molecules previously bound to β-CD. Prior research has highlighted that the primary driving force for complexation is the displacement of cavity-bound water molecules by hydrophobic guest molecules, as the latter form more stable interactions with the hydrophobic interior of β-CD [49]. During the encapsulation process, hydrophobic compounds in taro essence replaced water molecules initially bound within the β-CD cavity, altering the thermal decomposition pattern of β-CD. As shown in Figure 6, the weight loss rate of taro essence microcapsules became lower than that of β-CD at 313 °C, blank microcapsules at 336 °C, and taro essence alone at 355 °C. This delayed decomposition is attributed to the strong interactions between taro essence guest molecules and β-CD host molecules, enhancing the thermal stability of the encapsulated taro essence.

### 3.6. DSC Analysis of Taro Essence Microcapsules

**Figure 7:**
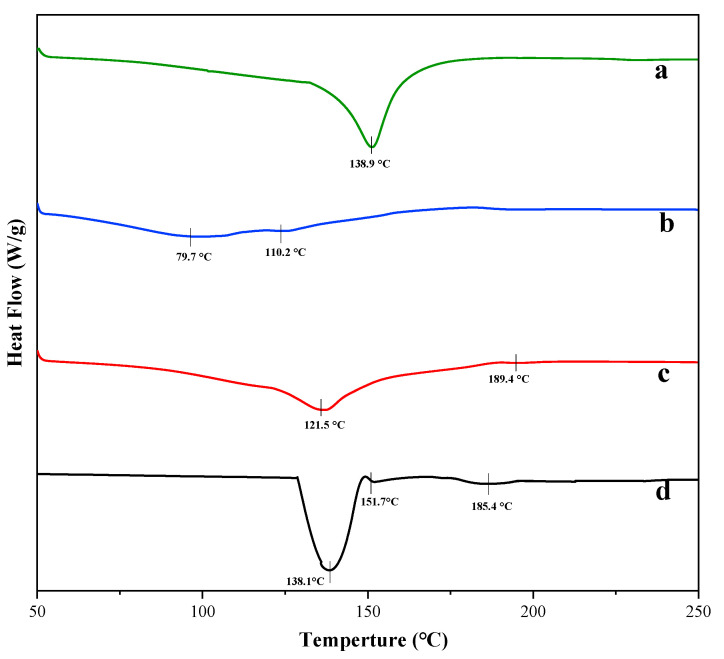
The DSC curves of β-CD (**a**), blank microcapsules (**b**), taro essence microcapsules, (**c**) and taro essence (**d**).

The DSC analysis was conducted to elucidate the thermal characteristics of taro essence microcapsules. The DSC thermograms presented in Figure 7 provide insightful information regarding the thermal behavior of the samples. Notably, the microcapsules exhibited distinct endothermic peaks, indicating a pronounced protective effect on the taro essence. For β-CD, a prominent endothermic peak was observed at 138.9 °C, likely associated with the loss of water molecules within the β-CD cavity [50,51]. The shift of the peak to lower temperatures and its broadening in blank microcapsules serve as evidence for the reduction in enthalpy (ΔH) and the disruption of water molecules within the CD cavity, possibly due to the oxidation or evaporation of some water molecules during the encapsulation process upon heating [52]. The DSC curve of the taro essence revealed three endothermic peaks at 138.1 °C, 151.7 °C, and 185.4 °C, which may correspond to water evaporation and the volatilization of fragrance components, respectively [53]. Following encapsulation, notable changes were observed in the DSC profile of the microcapsules. The shift in the endothermic peaks and their reduced areas in the microcapsules reflect the disordering of water molecules within the β-CD cavity [54]. Notably, the typical endothermic peak of β-CD was absent in the microcapsule curve, suggesting a deviation from the thermal behavior of pure β-CD and its blank microcapsules. The taro essence microcapsules exhibited a relatively higher temperature profile, with the peak temperature of the second endothermic peak surpassing that of the first and second peaks of the pure essence, underscoring the successful protection of the essence within the hydrophobic cavity of β-CD. Additionally, the absence and displacement of absorption peaks in the essence after encapsulation serve as compelling evidence for the formation of inclusion complexes [54]. These findings collectively demonstrate the efficacy of the microencapsulation process in enhancing thermal stability and protecting the volatile components of the taro essence.

### 3.7. FT-IR Analysis of Taro Essence Microcapsules

**Figure 8:**
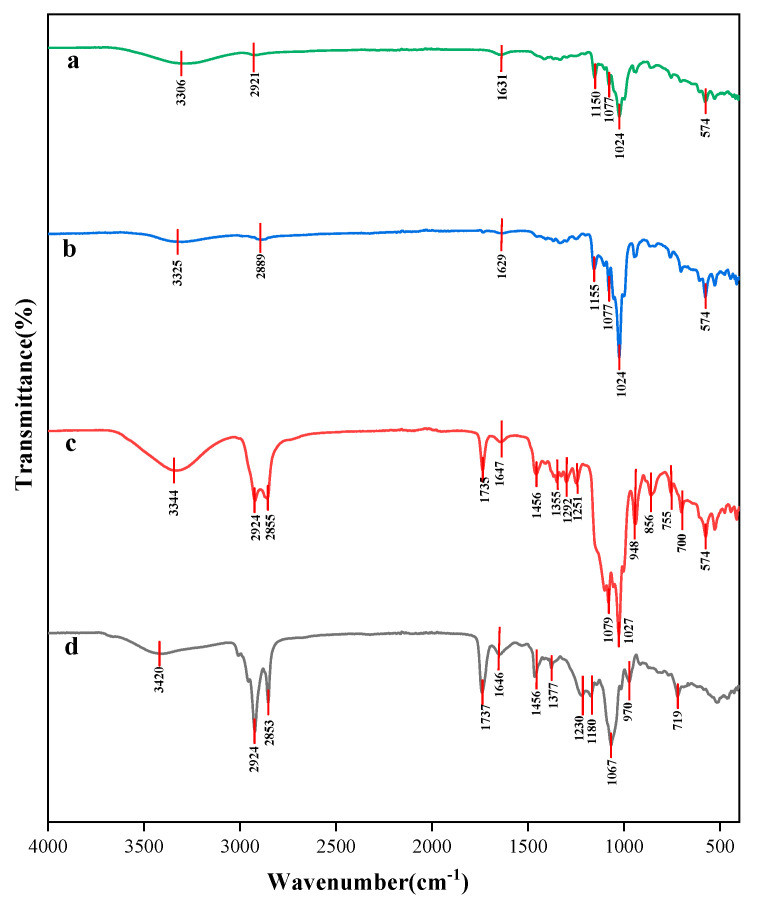
The FT-IR spectra of β-CD (**a**), blank microcapsules (**b**), taro essence microcapsules, (**c**) and taro essence (**d**).

FTIR spectroscopy is commonly employed to characterize interactions between guest molecules and cyclodextrins [55]. As depicted in Figure 8, the infrared spectra of β-CD, blank microcapsules, and microcapsules encapsulating taro essence were presented. The spectra of β-CD and blank microcapsules were essentially consistent, as shown in Figure 8a and Figure 8b, respectively. The FTIR spectrum aligned well with those reported in the literature for β-CD molecules [56], featuring peaks at 3306 cm^−1^, 2921 cm^−1^, 1631 cm^−1^, and 1024 cm^−1^, which were attributed to the symmetric stretching vibration of -OH groups, C-H stretching vibrations, H-O-H bending vibrations, and asymmetric C-O-C stretching vibrations, respectively, within the β-CD molecule. Subsequent volatile component analysis revealed the presence of alcohols, aldehydes, alkanes, and alkenes among the organic constituents of taro essence. In Figure 8d, a broad peak at 3420 cm^−1^ corresponded to the stretching vibration of -OH groups in the taro essence components, while peaks at 2924 cm^−1^ and 2853 cm^−1^ were indicative of alkyl C-H stretching vibrations. Furthermore, peaks at 1737 cm^−1^ and 1646 cm^−1^ were associated with the stretching vibrations of carbonyl C=O and olefinic C=C bonds, respectively, present in the essence. In contrast, Figure 8c revealed that, compared with the blank microcapsules, the microcapsules encapsulating taro essence exhibited characteristic peaks at 2924 cm^−1^, 2855 cm^−1^, 1735 cm^−1^, and 1647 cm^−1^, which were attributed to the taro essence components. This observation signified the successful encapsulation of taro essence within the microcapsules. Notably, the strongest and most rounded peak observed at 3344 cm^−1^ suggested the presence of intermolecular hydrogen bonding [57], implying a robust intermolecular interaction between the taro essence and β-CD. This robust interaction, coupled with their excellent biocompatibility, facilitated the effective inclusion of taro essence within the β-CD matrix.

### 3.8. Morphological Analysis of Taro Essence Microcapsules by SEM

**Figure 9:**
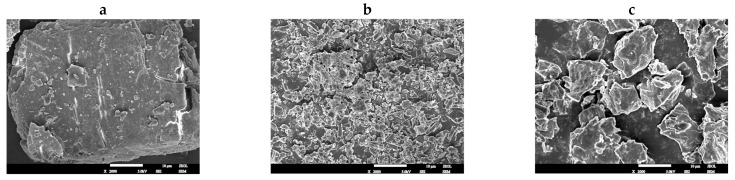
The SEM images of β-CD (**a**), blank microcapsules (**b**), and taro essence microcapsules (**c**).

SEM utilized a focused beam of high-energy electrons to scan samples, ultimately producing a clear image of the surface topography of the specimen [58,59]. This capability made SEM invaluable for visually evaluating the surface morphology of microcapsules, encompassing attributes such as shape, size, uniformity, and surface roughness. Figure 9 showcased SEM images of β-CD, blank microcapsules and flavor microcapsule powders that had been obtained. The results showed that β-CD exhibited a bulky, irregularly shaped block-like structure. In contrast, blank microcapsules displayed fragmented, block-like crystal structures with smaller sizes. Lacking an encapsulated core material, these blank microcapsules might have a more uniform morphology but lacked specific functional characteristics. The taro essence microcapsules displayed an irregular, heterogeneous morphology, characterized by varying sizes and a few grooves and wrinkles on their surfaces. These features could be attributed to the uneven internal pressure distribution within the microcapsules resulting from the inclusion of the taro essence, as well as interactions between the essence and the wall material. These grooves and wrinkles not only augmented the specific surface area of the microcapsules but potentially influenced their release properties and stability [60]. Additionally, the volatility of the essence may have contributed to certain surface modifications following microencapsulation.

### 3.9. XRD Analysis of Taro Essence Microcapsules

**Figure 10:**
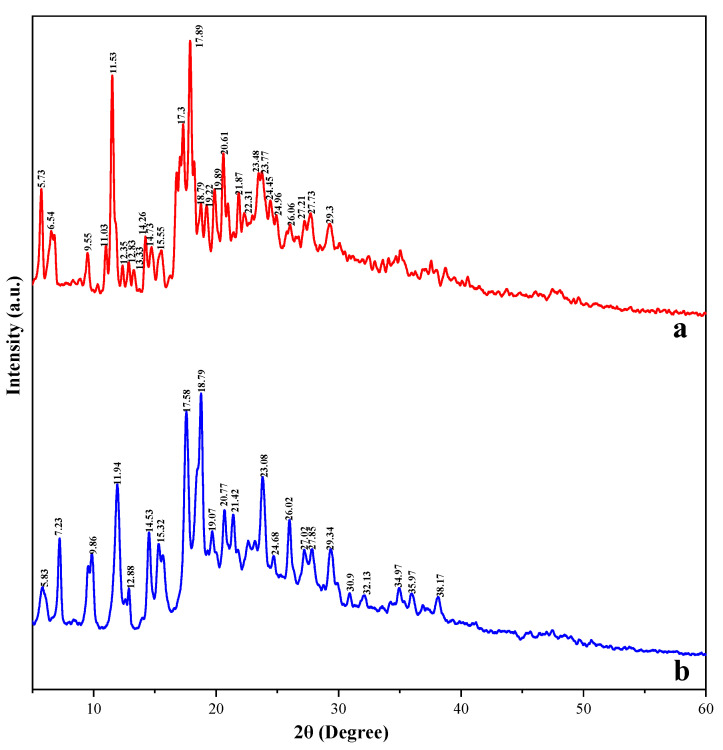
The XRD patterns of blank microcapsules (**a**) and taro essence microcapsules (**b**).

XRD has proven to be an invaluable tool in elucidating the formation of inclusion complexes, as evidenced by changes in crystallographic reflection peaks across various sample spectra, indicative of such complexation [61]. Figure 10 shows the XRD patterns of both blank microcapsules and taro flavor essence microcapsules, displaying intricate spectral line characteristics within their crystalline frameworks. The XRD pattern for the blank microcapsules revealed distinct and sharp characteristic peaks at 2θ diffraction angles of 5.73°, 11.53°, 17.3°, 17.89°, and 20.61°; minor peaks were identified at 9.55°, 14.26°, 19.89°, 21.87°, and 29.3°. In contrast, the XRD pattern of the taro flavor essence microcapsules exhibited a reduction in intensity and slight deviations in the positions of the characteristic peaks originating from the blank microcapsules, particularly at 5.83°, 11.53°, 17.89°, and 20.61°. Notably, the appearance of novel characteristic peaks at 7.23°, 17.58°, and 23.08° signifies the integration of new components. The XRD analysis conclusively confirmed that during the microencapsulation process, the taro flavor was successfully entrapped within the cavities of β-CD, leading to the formation of stable complexes.

### 3.10. Analysis of Volatile Flavor Components in Taro Essence Microcapsules

**Figure 11:**
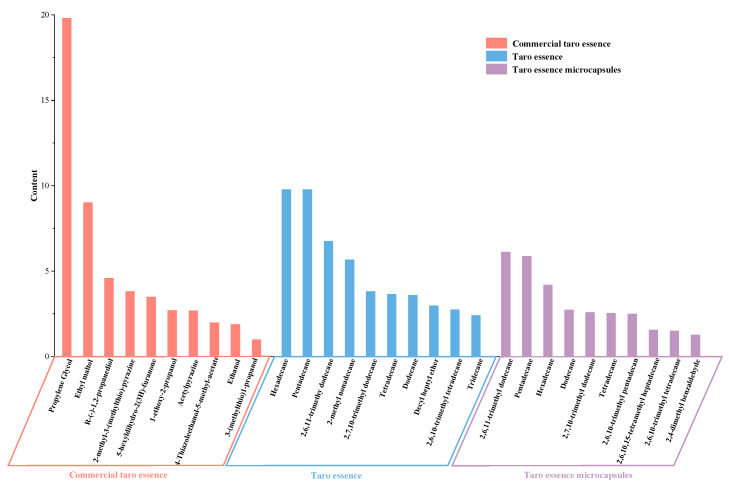
The top 10 volatile flavor components of commercial taro essence, taro essence, and its microcapsules.

Microencapsulation holds a pivotal position in safeguarding and stabilizing volatile components, such as essence and fragrances, which are indispensable across diverse industries encompassing food, pharmaceuticals, and cosmetics [8,62]. This encapsulation methodology bolsters the stability and controlled liberation of volatile compounds, thereby prolonging their shelf life and safeguarding their sensory attributes [63]. Consequently, analyzing taro flavor is imperative for assessing the efficacy of micro-encapsulation techniques and refining production methodologies to enhance the delivery and sensory impact of volatile components in end products. HS-SPME-GCMS analysis identified and quantified volatile components in taro essence microcapsules, with detailed composition and quantities listed in Supplementary Table S1. The analysis of taro flavor essence revealed the presence of 69 volatile compounds, including 37 alkanes, 11 alcohols, 6 aldehydes, 5 esters, 4 alkenes, 3 ketones, 2 phenols, and 1 ether. Microencapsulated taro essence retained 31 volatiles, with 20 alkanes remaining predominant, followed by aldehydes (4), alcohols (3), esters (2), alkenes (1), and ketones (1). Notably, key flavor-associated alkanes, including hexadecane, pentadecane, and 2,6,11-trimethyl dodecane, were successfully encapsulated (Figure 11), suggesting that cyclodextrin-based microencapsulation stabilized hydrophobic compounds during processing.

In contrast, the volatile components of commercial taro essence analyzed (see Supplementary Table S2), which primarily consisted of alcohols (12), alkanes (6), ketones (4), aldehydes (4), pyrazines (4), acids (4), phenols (1), esters (1), alkenes (1), and other compounds (5). The analysis revealed that commercial flavoring is predominantly composed of alcohols, with six out of its top ten components belonging to this category, showing a clear distinction from the natural taro essence. As shown in Figure 11, the three most abundant components in commercial flavoring were propylene glycol, ethyl maltol, and R-(-)-1,2-propanediol, all of which are synthetic additives. Propylene glycol functions as an emulsifier and stabilizer, ethyl maltol serves as a flavor enhancer, and R-(-)-1,2-propanediol is a chiral isomer of propylene glycol. Additionally, ethanol, ranked ninth, is used as an organic solvent and may leave residual traces. These additives not only alter the natural taro flavor profile but also pose potential health concerns. Therefore, this study employs microencapsulation technology to stabilize and protect natural taro essence, reducing the reliance on synthetic additives and providing a flavoring solution that aligns with clean-label requirements.

### 3.11. Analysis of Volatile Flavor Components in Taro Essence Microcapsules During Storage

**Figure 12:**
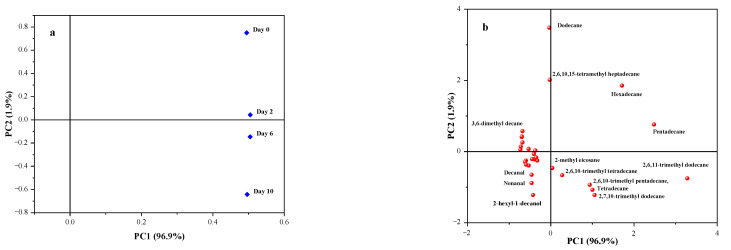
Principal component scores (**a**) and loading plot (**b**) of taro essence microcapsules during the storage.

Principal Component Analysis (PCA) is a potent statistical approach for dimensionality reduction and the insightful exploration of extensive datasets [64]. It adeptly uncovered latent patterns and correlations within the data, as evidenced in the analysis of volatile compounds during microcapsule storage. The PCA score plot in Figure 12 revealed the distribution of PC1 and PC2 across distinct quadrants, highlighting commonalities and contrasting differences in the flavor substances volatilized at various stages. The volatile components remained in the same quadrant on day 2 as observed on day 0. However, by day 6, the components had shifted to the fourth quadrant, reflecting a discernible alteration. This shift became more pronounced after 10 days of storage, demonstrating a significant divergence from the initial state. The distance of sample data points from the center in the load map directly correlates with the importance of the corresponding substances, with farther points indicating higher significance, and specific compounds demonstrating strong correlations in different principal components [65,66]. The loading plot showed that most substances were concentrated towards the center of the plot, with notable exceptions including dodecane, 2,6,10,15-tetramethyl heptadecane, hexadecane, pentadecane, and 2,6,11-trimethyl dodecane. Notably, 2,6,11-trimethyl dodecane and pentadecane exhibited a strong positive correlation with PC1; dodecane, 2,6,10,15-tetraethyl heptadecane showed a high positive correlation in PC2; while hexadecane showed a significant positive correlation in PC1 and PC2, indicating their abundance and high importance during this period. This analysis aided in visualizing the variable relationships between flavor components and samples, providing insights into the temporal changes and significance of volatile compounds during storage.

**Figure 13:**
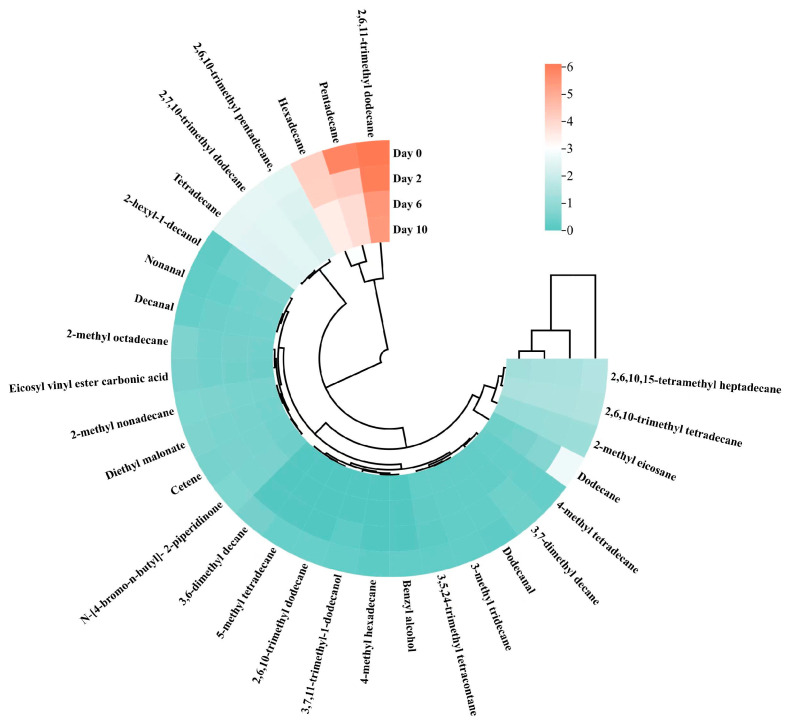
Clustering and heatmap analysis of volatile flavor compounds in taro essence microcapsules during the storage.

The heatmap clustering analysis, a widely employed technique for comparing variations among essence constituents, effectively unveils data patterns by grouping similar entities based on proximity metrics [67]. This approach was applied to track changes in the components of taro essence microcapsules over a 10-day storage period. As shown in Figure 13, the volatile flavor components within the microcapsules exhibited minimal changes in concentration over time. The heatmap clearly illustrated the stability of most volatile substances. However, pentadecane experienced a marked decrease on the second day of storage, followed by a gradual decline, while dodecane showed a significant reduction on the second day and remained stable thereafter. Hexadecane and 2,6,11-trimethyl dodecane exhibited slight decreases after six days of storage but stabilized until the 10th day. These trends are strongly correlated with PC1 and PC2, aligning with the PCA results. Microencapsulation plays a crucial role in protecting volatile flavor compounds from external factors such as temperature, light, and oxygen, which can degrade these components [8]. Additionally, previous studies have demonstrated that microcapsules containing phase-change materials can help maintain stable storage temperatures for food products, underscoring the versatile applications of microencapsulation in preserving flavors and aromas [68]. The data underscore the importance of monitoring and understanding the evolution of taro essence volatile components during storage to ensure product quality and stability.

## 4. Conclusions

This research comprehensively analyzed the effects of core-to-wall ratio, T-20/β-CD mass ratio, and ultrasonic time on the encapsulation efficiency of taro essence microcapsules. Orthogonal experiments further refined these parameters, identifying an optimal combination with a core-to-wall ratio of 1:10, a T-20/β-CD mass ratio of 1.6:1, and an ultrasonic time of 40 min that resulted in an embedding efficiency of 56.10%. The microcapsules exhibited desirable physical properties, including appropriate moisture content, solubility, bulk density, and flowability, ensuring stability and functionality. Particle size analysis showed uniform distribution, while zeta potential measurements indicated good stability against agglomeration. Thermal analysis (TGA and DSC) confirmed improved thermal stability, and FT-IR analysis validated successful encapsulation with strong intermolecular interactions between taro essence and β-CD. SEM images revealed the heterogeneous morphology of the microcapsules, influenced by the encapsulated essence. XRD patterns indicated the formation of stable inclusion complexes. The analysis of volatile components highlighted the successful encapsulation of key alkanes, with PCA and heatmap clustering providing insights into the stability and evolution of these components during storage. Overall, this study demonstrated the efficacy of the optimized microencapsulation process in enhancing the encapsulation efficiency, stability, and thermal properties of taro essence microcapsules. However, the EE still has limitations and requires further improvement. Our future research will focus on optimizing encapsulation techniques and expanding the application of natural taro essence microcapsules in food processing.

## Figures and Tables

**Table 1:** Table of factors and levels.

Title 1	Factors		
	A (Taro Flavor/β-CD)	B (T-20/β-CD)	C (Ultrasound Time)
1	1:8	1.2:1	30
2	1:10	1.4:1	40
3	1:12	1.6:1	50

**Table 2:** Results and analysis of orthogonal experiments.

NO.	A	B	C	EE (%)
1	1	1	1	36.38 ± 0.03
2	1	2	2	46.70 ± 0.01
3	1	3	3	48.92 ± 0.05
4	2	1	2	55.94 ± 0.01
5	2	2	3	52.29 ± 0.04
6	2	3	1	49.84 ± 0.01
7	3	1	3	49.37 ± 0.01
8	3	2	1	52.45 ± 0.01
9	3	3	2	54.72 ± 0.01
K1	44.030	47.230	46.223	
K2	52.690	50.510	52.483	
K3	52.180	51.160	50.193	
R	8.660	3.930	6.260	
Significance order of factors		ACB		
Optimal preparation condition		A_2_B_3_C_2_		

**Table 3:** The basic physical properties of taro essence microcapsules.

NO.	Physical Propezrties	Unit	Value
1	Moisture content	%	4.26 ± 0.04
2	Solubility	%	61.50 ± 0.05
3	Angle of repose	°	40.49 ± 0.34
4	*ρ*_a_	mg/mL	0.04 ± 0.004
5	*ρ*_b_	mg/mL	0.04 ± 0.006
6	CI	-	12.72 ± 0.40
7	HR	-	0.88 ± 0.003

## Data Availability

The data supporting the findings of this study are available from the corresponding author upon request.

## Supplementary Materials

The following supporting information can be downloaded at: https://www.mdpi.com/article/10.3390/foods14050754/s1, Table S1: Volatile flavor components in taro essence and taro essence microcapsules; Table S2: Volatile flavor compounds in commercial taro essence.

## References

[1] Zhang E., Shen W., Jiang W., Li W., Wan X., Yu X., Xiong F. (2023). Research Progress on the Bulb Expansion and Starch Enrichment in Taro *(Colocasia esculenta* (L). Schott). PeerJ.

[2] Zubair M.W., Imran A., Islam F., Afzaal M., Saeed F., Zahra S.M., Akhtar M.N., Noman M., Ateeq H., Aslam M.A. (2023). Functional Profile and Encapsulating Properties of *Colocasia esculenta* (Taro). Food Sci. Nutr..

[3] Nip W.-K. (2023). Taro. Processing Vegetables.

[4] Muharja M., Darmayanti R.F., Khamil A.I., Prastika A., Rizalluddin M., Fadilah S.N., Sari D.A.D. (2023). Evaluation of Dehydration Performance of Belitung Taro (Xanthosoma Sagittifolium) Using Tray Dryer. J. Technol. Sci..

[5] Nurilmala F., Masnang A., Sonani N. (2023). Pendampingan Budidaya Talas Varietas Baru Bagi KWT Sawargi Kelurahan Situgede Kecamatan Bogor Barat Kota Bogor. J. Abdimas Adpi Sos. Hum..

[6] Sousa V.I., Parente J.F., Marques J.F., Forte M.A., Tavares C.J. (2022). Microencapsulation of Essential Oils: A Review. Polymers.

[7] Bakry A.M., Abbas S., Ali B., Majeed H., Abouelwafa M.Y., Mousa A., Liang L. (2016). Microencapsulation of Oils: A Comprehensive Review of Benefits, Techniques, and Applications. Comp. Rev. Food Sci. Food Safe.

[8] Kłosowska A., Wawrzyńczak A., Feliczak-Guzik A. (2023). Microencapsulation as a Route for Obtaining Encapsulated Flavors and Fragrances. Cosmetics.

[9] Russell S., Bruns N. (2023). Encapsulation of Fragrances in Micro- and Nano-Capsules, Polymeric Micelles, and Polymersomes. Macromol. Rapid Commun..

[10] El Kharraf S., Farah A., El-Guendouz S., Lourenço J.P., Rosa Costa A.M., El Hadrami E.M., Machado A.M., Tavares C.S., Figueiredo A.C., Miguel M.G. (2023). *β*-Cyclodextrin Inclusion Complexes of Combined Moroccan *Rosmarinus officinalis*, *Lavandula angustifolia* and *Citrus aurantium* Volatile Oil: Production Optimization and Release Kinetics in Food Models. J. Essent. Oil Res..

[11] Liu H.-N., Jiang X.-X., Naeem A., Chen F.-C., Wang L., Liu Y.-X., Li Z., Ming L.-S. (2022). Fabrication and Characterization of β-Cyclodextrin/Mosla Chinensis Essential Oil Inclusion Complexes: Experimental Design and Molecular Modeling. Molecules.

[12] Iliadi E., Lamari F. (2022). Encapsulation of Two Distinct Cistus Essential Oils in β- and γ-Cyclodextrins. Planta Medica.

[13] Albuquerque P.M., Azevedo S.G., De Andrade C.P., D’Ambros N.C.D.S., Pérez M.T.M., Manzato L. (2022). Biotechnological Applications of Nanoencapsulated Essential Oils: A Review. Polymers.

[14] Vasisht N. (2023). Factors and Mechanisms in Microencapsulation. Microencapsulation in the Food Industry.

[15] Tian Y., Luo W., Wang Y., Yu Y., Huang W., Tang H., Zheng Y., Liu Z. (2021). Ultrasound-Assisted Fast Encapsulation of Metal Microparticles in SiO_2_ via an Interface-Confined Sol-Gel Method. Ultrason. Sonochem..

[16] Zhu H., Zhang Y., Tian J., Chu Z. (2018). Effect of a New Shell Material—Jackfruit Seed Starch on Novel Flavor Microcapsules Containing Vanilla Oil. Ind. Crops Prod..

[17] Santos F.S.D., Figueirêdo R.M.F.D., Queiroz A.J.D.M., Paiva Y.F., Moura H.V., Silva E.T.D.V., Ferreira J.P.D.L., Melo B.A.D., Carvalho A.J.D.B.A., Lima M.D.S. (2023). Influence of Dehydration Temperature on Obtaining Chia and Okra Powder Mucilage. Foods.

[18] Quispe-Condori S., Saldaña M.D., Temelli F. (2011). Microencapsulation of Flax Oil with Zein Using Spray and Freeze Drying. LWT.

[19] Nishad J., Selvan C.J., Mir S.A., Bosco S.J.D. (2017). Effect of Spray Drying on Physical Properties of Sugarcane Juice Powder (*Saccharum officinarum* L.). J. Food Sci. Technol..

[20] Helgason T., Awad T.S., Kristbergsson K., McClements D.J., Weiss J. (2009). Effect of Surfactant Surface Coverage on Formation of Solid Lipid Nanoparticles (SLN). J. Colloid Interface Sci..

[21] Sharma R., Borah A., Sharma R., Borah A. (2021). Prospect of Microcapsules as a Delivery System in Food Technology: A Review. Pharma Innov. J..

[22] da Dias D.R.C., Barros Z.M.P., de Carvalho C.B.O., Honorato F.A., Guerra N.B., Azoubel P.M. (2015). Effect of Sonication on Soursop Juice Quality. LWT.

[23] Chen Q., Chen Y., Xu Q., Jin H., Hu Q., Han D. (2022). Effective Two-Stage Heterotrophic Cultivation of the Unicellular Green Microalga Chromochloris Zofingiensis Enabled Ultrahigh Biomass and Astaxanthin Production. Front. Bioeng. Biotechnol..

[24] Xu S., Bi J., Jin W., Fan B., Qian C. (2022). Determination of Polysaccharides Composition in Polygonatum Sibiricum and Polygonatum Odoratum by HPLC-FLD with Pre-Column Derivatization. Heliyon.

[25] Lengyel M., Kállai-Szabó N., Antal V., Laki A.J., Antal I. (2019). Microparticles, Microspheres, and Microcapsules for Advanced Drug Delivery. Sci. Pharm..

[26] Nolazco–Cama D., Sánchez-Contreras A., Tellez-Monzón L., Vargas-Delgado L., Condezo-Hoyos L. (2023). Influence of Essential Oil: Cyclodextrin Ratio and Stirring Rate on Physicochemical Characteristics of Orange Essential Oil: β-Cyclodextrin Microparticles. CyTA-J. Food.

[27] Otálora M.C., Wilches-Torres A., Gómez Castaño J.A. (2023). Microencapsulation of Betaxanthin Pigments from Pitahaya (*Hylocereus megalanthus*) by-Products: Characterization, Food Application, Stability, and in Vitro Gastrointestinal Digestion. Foods.

[28] Yeddes W., Mejri I., Wannes W.A., Affes T.G., Khammassi S., Hammami M., Tounsi M.S. (2022). Effect of Emulsifiers and Wall Materials on Particle Size Distribution and Stability of the Blended Essential Oils Nanoemulsions. J. Sustain. Mater. Process. Manag..

[29] Ravindran Maniam M.M., Loong Y.H., Samsudin H. (2022). Understanding the Formation of β-Cyclodextrin Inclusion Complexes and Their Use in Active Packaging Systems. Starch Stärke.

[30] Lad S., Narkhede S., Luhar S., Prajapati A. (2022). Review on Moisture Content: A Stability Problem in Pharmaceuticals. EPRA Int. J. Res. Dev. (IJRD).

[31] Biyani M. (2021). HPMC Capsules for Moisture Sensitive and Hygroscopic Products. Pharm. Sci. Technol..

[32] Sukri N., Annisa D.S., Djali M., Cahyana Y., Mahani M., Huda S. (2023). Effect of Whey Protein Isolate (WPI)–Pectin Ratio on Phenolic Content Stability of Propolis Microcapsules. Preprints.

[33] Nasr A.M., Elhady S.S., Swidan S.A., Badawi N.M. (2020). Celecoxib Loaded In-Situ Provesicular Powder and Its In-Vitro Cytotoxic Effect for Cancer Therapy: Fabrication, Characterization, Optimization and Pharmacokinetic Evaluation. Pharmaceutics.

[34] Carneiro H.C., Tonon R.V., Grosso C.R., Hubinger M.D. (2013). Encapsulation Efficiency and Oxidative Stability of Flaxseed Oil Microencapsulated by Spray Drying Using Different Combinations of Wall Materials. J. Food Eng..

[35] Sahoo N., Sahoo R.K., Biswas N., Guha A., Kuotsu K. (2015). Recent Advancement of Gelatin Nanoparticles in Drug and Vaccine Delivery. Int. J. Biol. Macromol..

[36] Saifullah M., Yusof Y.A., Chin N.L., Aziz M.G. (2016). Physicochemical and Flow Properties of Fruit Powder and Their Effect on the Dissolution of Fast Dissolving Fruit Powder Tablets. Powder Technol..

[37] Benyerbah N., Ispas-Szabo P., Sakeer K., Chapdelaine D., Mateescu M.A. (2019). Ampholytic and Polyelectrolytic Starch as Matrices for Controlled Drug Delivery. Pharmaceutics.

[38] Mahmoud E.A., Bendas E.R., Mohamed M.I. (2009). Preparation and Evaluation of Self-Nanoemulsifying Tablets of Carvedilol. AAPS PharmSciTech.

[39] Li Y., Zou Y., Que F., Zhang H. (2022). Recent Advances in Fabrication of Edible Polymer Oleogels for Food Applications. Curr. Opin. Food Sci..

[40] Tomsone L., Galoburda R., Kruma Z., Durrieu V., Cinkmanis I. (2020). Microencapsulation of Horseradish (*Armoracia rusticana* L.) Juice Using Spray-Drying. Foods.

[41] Shi Y., Wang S., Tu Z., Wang H., Li R., Zhang L., Huang T., Su T., Li C. (2016). Quality Evaluation of Peony Seed Oil Spray-Dried in Different Combinations of Wall Materials during Encapsulation and Storage. J. Food Sci. Technol..

[42] Jornada D.S., Fiel L.A., Bueno K., Gerent J.F., Petzhold C.L., Beck R.C., Guterres S.S., Pohlmann A.R. (2012). Lipid-Core Nanocapsules: Mechanism of Self-Assembly, Control of Size and Loading Capacity. Soft Matter.

[43] Zulkefli N.N., Seladorai R., Masdar M.S., Mohd Sofian N., Wan Isahak W.N.R. (2022). Core Shell Nanostructure: Impregnated Activated Carbon as Adsorbent for Hydrogen Sulfide Adsorption. Molecules.

[44] Cheng T., Chen H., Wei Q. (2022). The Role of Roller Rotation Pattern in the Spreading Process of Polymer/Short-Fiber Composite Powder in Selective Laser Sintering. Polymers.

[45] Liu T., Gao Z., Zhong W., Fu F., Li G., Guo J., Shan Y. (2022). Preparation, Characterization, and Antioxidant Activity of Nanoemulsions Incorporating Lemon Essential Oil. Antioxidants.

[46] Gulzar S., Nilsuwan K., Raju N., Benjakul S. (2022). Whole Wheat Crackers Fortified with Mixed Shrimp Oil and Tea Seed Oil Microcapsules Prepared from Mung Bean Protein Isolate and Sodium Alginate. Foods.

[47] Shi Y., Fu L., Chen X., Guo J., Yang F., Wang J., Zheng Y., Hu Y. (2017). Hypophosphite/Graphitic Carbon Nitride Hybrids: Preparation and Flame-Retardant Application in Thermoplastic Polyurethane. Nanomaterials.

[48] Smith V.J., Caira M.R., Hunter R., Bourne S.A. (2004). Characterization of (E)- and (Z)-Ajoene Inclusion Complexes with α-, β-, γ- and Permethylated-β-Cyclodextrin Using PXRD, Single-Crystal X-Ray Diffraction and Thermal Analysis. Acta Crystallogr. Sect. A.

[49] Dos Santos C., Buera M.P., Mazzobre M.F. (2011). Phase Solubility Studies and Stability of Cholesterol/Β-cyclodextrin Inclusion Complexes. J. Sci. Food Agric..

[50] Su J., Chen J., Li L., Li B., Shi L., Chen L., Xu Z. (2012). Formation of β-Cyclodextrin Inclusion Enhances the Stability and Aqueous Solubility of Natural Borneol. J. Food Sci..

[51] Wang J., Cao Y., Sun B., Wang C. (2011). Physicochemical and Release Characterisation of Garlic Oil-β-Cyclodextrin Inclusion Complexes. Food Chem..

[52] Kfoury M., Auezova L., Greige-Gerges H., Ruellan S., Fourmentin S. (2014). Cyclodextrin, an Efficient Tool for Trans-Anethole Encapsulation: Chromatographic, Spectroscopic, Thermal and Structural Studies. Food Chem..

[53] Li J., Hou X., Jiang L., Xia D., Chen A., Li S., Li Q., Gu X., Mo X., Zhang Z. (2022). Optimization and Characterization of Sichuan Pepper (Zanthoxylum Bungeanum Maxim) Resin Microcapsule Encapsulated with β-Cyclodextrin. LWT.

[54] Zhang W., Li X., Yu T., Yuan L., Rao G., Li D., Mu C. (2015). Preparation, Physicochemical Characterization and Release Behavior of the Inclusion Complex of Trans-Anethole and β-Cyclodextrin. Food Res. Int..

[55] Mura P. (2015). Analytical Techniques for Characterization of Cyclodextrin Complexes in the Solid State: A Review. J. Pharm. Biomed. Anal..

[56] Yan Y., Zhao X., Wang C., Fang Q., Zhong L., Wei Q. (2022). Preparation, Optimization, and Characterization of Inclusion Complexes of Cinnamomum Longepaniculatum Essential Oil in β-Cyclodextrin. Sustainability.

[57] Zhang L., Wang X., He Y., Cao J., Wang K., Lin H., Qu C., Miao J. (2022). Regulatory Effects of Functional Soluble Dietary Fiber from Saccharina Japonica Byproduct on the Liver of Obese Mice with Type 2 Diabetes Mellitus. Mar. Drugs.

[58] Topper T.P., Guo J., Clausen S., Skovsted C.B., Zhang Z. (2020). Reply to ‘Re-Evaluating the Phylogenetic Position of the Enigmatic Early Cambrian Deuterostome Yanjiahella’. Nat. Commun..

[59] Joo K.-N., Park H.-M. (2022). Recent Progress on Optical Tomographic Technology for Measurements and Inspections of Film Structures. Micromachines.

[60] Ma Z., Liao H., Pan Z., Cheng F. (2022). Insights into Coproduction of Silica Gel via Desulfurization of Steel Slag and Silica Gel Adsorption Performance. ACS Omega.

[61] Abarca R.L., Rodríguez F.J., Guarda A., Galotto M.J., Bruna J.E. (2016). Characterization of Beta-Cyclodextrin Inclusion Complexes Containing an Essential Oil Component. Food Chem..

[62] Yin Y., Su X., Cadwallader K.R. (2023). Testing Tools and Physical, Chemical, and Microbiological Characterization of Microencapsulated Systems. Microencapsulation in the Food Industry.

[63] Saroch P., Naman S., Baldi A., Balakrishnan P., Gopi S. (2022). Delivery Systems for Flavors and Fragrances: Quality by Design-Based Considerations. ACS Symposium Series.

[64] Marzban C., Yurtsever U., Richman M. (2024). Principal Component Analysis for Equation Discovery. arXiv.

[65] Li C., Al-Dalali S., Wang Z., Xu B., Zhou H. (2022). Investigation of Volatile Flavor Compounds and Characterization of Aroma-Active Compounds of Water-Boiled Salted Duck Using GC–MS–O, GC–IMS, and E-Nose. Food Chem..

[66] Li C., Al-Dalali S., Zhou H., Wang Z., Xu B. (2021). Influence of Mixture of Spices on Phospholipid Molecules during Water-Boiled Salted Duck Processing Based on Shotgun Lipidomics. Food Res. Int..

[67] Lu W., Chen J., Li X., Qi Y., Jiang R. (2023). Flavor Components Detection and Discrimination of Isomers in Huaguo Tea Using Headspace-Gas Chromatography-Ion Mobility Spectrometry and Multivariate Statistical Analysis. Anal. Chim. Acta.

[68] Güngör Ertuğral T., Danışman M., Oral A. (2023). Microencapsulation of N-Tridecane/n-Tetradecane Eutectic Mixture with Poly (Methyl Methacrylate) Shell for Candidate for Food Packaging Thermal Energy Storage Material. Polym.-Plast. Technol. Mater..

