# Supplementary material for: Optimization and Comprehensive Characterization of the Microencapsulation Process for Taro Essence
Source: Foods. 2025 Feb 23;14(5):754. doi: 10.3390/foods14050754 (PMC11899469; doi:10.3390/foods14050754)
Supplement: Supplementary file 1 [file foods-14-00754-s001.zip › foods-3460813-supplementary.pdf]

Supplementary Material

Table S1. Volatile flavor components in taro essence and taro essence microcapsules.

NO.	Type	Name	Formula	CAS#	Compound content (μg/g)	
					Taro essence	Taro essence microcapsules
A1	alkane	1-methyl-2-octyl cyclopropane	$C_{12}H_{24}$	37617-26-8	0.19	-
A2	alkane	Dodecane	$C_{12}H_{26}$	112-40-3	3.59	2.72
A3	alkane	2-methyl decane	$C_{11}H_{24}$	6975-98-0	0.34	-
A4	alkane	Hexyl cyclopentane	$C_{11}H_{22}$	4457-00-5	0.19	-
A5	alkane	3,7-dimethyl decane	$C_{12}H_{26}$	17312-54-8	1.73	0.41
A6	alkane	3-methyl tridecane	$C_{14}H_{30}$	6418-41-3	0.25	0.22
A7	alkane	5-methyl tetradecane	$C_{15}H_{32}$	25117-32-2	0.20	0.36
A8	alkane	4-methyl tetradecane	$C_{15}H_{32}$	25117-24-2	0.29	0.28
A9	alkane	2,6,10-trimethyltridecane	$C_{16}H_{34}$	3891-99-4	1.54	-
A10	alkane	3,7-dimethyl undecane	$C_{13}H_{28}$	17301-29-0	0.52	-
A11	alkane	4,7-dimethyl undecane	$C_{13}H_{28}$	17301-32-5	0.12	-
A12	alkane	1-Iodo-2-methylundecane	$C_{12}H_{25}I$	73105-67-6	0.63	-
A13	alkane	Undecane, 3,6-dimethyl-	$C_{13}H_{28}$	17301-28-9	0.95	-
A14	alkane	Octane, 2,4,6-trimethyl-	$C_{11}H_{24}$	62016-37-9	0.33	-
A15	alkane	4-methyl hexadecane	$C_{17}H_{36}$	25117-26-4	0.45	0.15
A16	alkane	2,3,7-trimethyl decane	$C_{13}H_{28}$	62238-13-5	0.36	-
A17	alkane	2,6,10-trimethyl dodecane	$C_{15}H_{32}$	3891-98-3	2.18	0.37
A18	alkane	2,4-dimethyl eicosane	$C_{22}H_{46}$	75163-98-3	0.83	-
A19	alkane	5,5-Diethylheptadecane	$C_{21}H_{44}$	NA	0.58	-

A20	alkane	2,6,11-trimethyl dodecane	$C_{15}H_{32}$	31295-56-4	6.74	6.12
A21	alkane	2,7,10-trimethyl dodecane	$C_{15}H_{32}$	74645-98-0	3.80	2.58
A22	alkane	Tridecane	$C_{13}H_{28}$	629-50-5	2.39	-
A23	alkane	Tetradecane	$C_{14}H_{30}$	629-59-4	3.64	2.53
A24	alkane	2,6,10-trimethyl tetradecane	$C_{17}H_{36}$	14905-56-7	2.73	1.50
A25	alkane	Pentadecane	$C_{15}H_{32}$	629-62-9	9.77	5.87
A26	alkane	2,6,10-trimethyl pentadecane	$C_{18}H_{38}$	3892-00-0	1.46	2.49
A27	alkane	Hexadecane	$C_{16}H_{34}$	544-76-3	9.77	4.18
A28	alkane	2,6,10,15-tetramethyl heptadecane	$C_{21}H_{44}$	54833-48-6	1.18	1.55
A29	alkane	2-methyl octadecane	$C_{19}H_{40}$	1560-88-9	0.94	0.63
A30	alkane	3-ethyl-5-(2-ethylbutyl) octadecane	$C_{26}H_{54}$	55282-12-7	0.73	-
A31	alkane	2-methyl nonadecane	$C_{20}H_{42}$	1560-86-7	5.66	0.66
A32	alkane	2-methyl eicosane	$C_{21}H_{44}$	1560-84-5	1.38	1.16
A33	alkane	3,5,24-trimethyl tetracontane	$C_{43}H_{88}$	55162-61-3	1.11	0.21
A34	alkane	3,6-dimethyl decane	$C_{12}H_{26}$	17312-53-7	0.41	0.46
A35	alkane	Undecane	$C_{11}H_{24}$	1120-21-4	0.21	-
A36	alkane	Eicosane	$C_{20}H_{42}$	112-95-8	0.28	-
A37	alkane	2,6-dimethyl undecane	$C_{13}H_{28}$	17301-23-4	0.23	-
B1	alkene	(E)-3-Octadecene	$C_{18}H_{36}$	7206-19-1	1.15	-
B2	alkene	1-tridecene	$C_{13}H_{26}$	2437-56-1	0.37	-
B3	alkene	Cetene	$C_{16}H_{32}$	629-73-2	0.24	0.54
B4	alkene	(E)-9-Octadecene	$C_{18}H_{36}$	7206-25-9	1.22	-
C1	alcohol	1-Butanol	$C_4H_{10}O$	71-36-3	0.57	-

C2	alcohol	Benzyl alcohol	C_7H_8O	100-51-6	0.14	0.20
C3	alcohol	Terpineol	$C_{10}H_{18}O$	8000-41-7	0.19	-
C4	alcohol	4-methyl-5-Thiazoleethanol	C_6H_9NOS	137-00-8	0.15	-
C5	alcohol	3,7,11-trimethyl-1-dodecanol	$C_{15}H_{32}O$	6750-34-1	0.77	0.26
C6	alcohol	2-methyl-1-hexadecanol	$C_{17}H_{36}O$	2490-48-4	0.27	-
C7	alcohol	2-Hexyl-1-octanol	$C_{14}H_{30}O$	19780-79-1	0.78	-
C8	alcohol	2-Methyl-1-undecanol	$C_{12}H_{26}O$	10522-26-6	0.88	-
C9	alcohol	Tert-hexadecanethiol	$C_{16}H_{34}S$	25360-09-2	0.19	-
C10	alcohol	2-hexyl-1-decanol	$C_{16}H_{34}O$	2425-77-6	0.27	0.16
C11	alcohol	Benzyl alcohol	C_7H_8O	100-51-6	0.14	-
D1	aldehyde	Nonanal	$C_9H_{18}O$	124-19-6	0.29	0.24
D2	aldehyde	Decanal	$C_{10}H_{20}O$	112-31-2	0.58	0.32
D3	aldehyde	2,4-dimethyl- benzaldehyde,	$C_9H_{10}O$	15764-16-6	0.71	1.26
D4	aldehyde	Dodecanal	$C_{12}H_{24}O$	112-54-9	0.27	0.18
D5	aldehyde	E-15-Heptadecenal	$C_{17}H_{32}O$	NA	0.76	-
D6	aldehyde	Benzaldehyde	C_7H_6O	100-52-7	0.30	-
E1	ester	Diethyl malonate	$C_7H_{12}O_4$	105-53-3	0.65	0.63
E2	ester	2,2,4-trimethyl-1,3-pentanedio- 2,2-dimethyl-1-(1-methylethyl)-1,3-propanediyl ester-2-methyl ropanoic acid	$C_{16}H_{30}O_4$	NA	0.65	-
E3	ester	octadecyl pentyl ester sulfurous acid	$C_{23}H_{48}O_3S$	NA	0.13	-
E4	ester	Butyl-1,2-Benzenedicarboxylic acid	$C_{20}H_{30}O_4$	84-78-6	0.39	-
E5	ester	eicosyl vinyl ester carbonic acid	$C_{23}H_{44}O_3$	NA	1.26	0.48
F1	ketones	(E,E)-3,5-Octadien-2-one	$C_8H_{12}O$	30086-02-3	0.73	-

F2	ketones	6,10,14-trimethyl-2-Pentadecanone	$C_{18}H_{36}O$	502-69-2	0.38	-
F3	ketones	N-[4-bromo-n-butyl]- 2-Piperidinone	$C_9H_{16}BrNO$	NA	0.90	0.65
G1	phenol	Maltol	$C_6H_6O_3$	118-71-8	0.38	-
G2	phenol	2,4-Di-tert-butylphenol	$C_{14}H_{22}O$	96-76-4	2.29	-
H	ether	Decyl heptyl ether	$C_{17}H_{36}O$	NA	2.97	-

Table S2 Volatile flavor compounds in commercial taro essence

NO.	Type	Name	Formula	CAS#	Compound content (μg/g)
A1	alcohol	Propylene glycol	$C_3H_8O_2$	57-55-6	19.79
A2	alcohol	R-(-)-1,2-propanediol	$C_3H_8O_2$	4254-14-2	4.58
A3	alcohol	1-ethoxy-2-Propanol	$C_5H_{12}O_2$	1569-02-4	2.69
A4	alcohol	Isopropyl Alcohol	C_3H_8O	67-63-0	0.07
A5	alcohol	Ethanol	C_2H_6O	64-17-5	1.88
A6	alcohol	3-(methylthio)-1-propanol	$C_4H_{10}OS$	505-10-2	0.98
A7	alcohol	2-Butanol	$C_4H_{10}O$	78-92-2	0.14
A8	alcohol	4-allyloxy-2-methyl-pentan-2-ol	$C_9H_{18}O_2$	102840-52-8	0.10
A9	alcohol	5-methyl-, acetate4-thiazoleethanol	$C_8H_{11}NO_2S$	NA	1.98
A10	alcohol	4-methyl- acetate1-hexanol	$C_9H_{18}O_2$	91367-59-8	0.04
A11	alcohol	2,2'-oxybis-1-propanol	$C_6H_{14}O_3$	108-61-2	0.03
A12	alcohol	(3-Methyl-oxiran-2-yl)-methanol	$C_4H_8O_2$	NA	0.01
B1	alkane	1,4,7,10,13,16-Hexaoxacyclooctadecane	$C_{12}H_{24}O_6$	17455-13-9	0.39
B2	alkane	2-methyl-decane	$C_{11}H_{24}$	6975-98-0	0.30
B3	alkane	Octadecane	$C_{18}H_{38}$	593-45-3	0.05
B4	alkane	2-methyl-nonadecane	$C_{20}H_{42}$	1560-86-7	0.03
B5	alkane	2-methyl-eicosane	$C_{21}H_{44}$	18344-37-1	0.02

B6	alkane	2,6,10,14-tetramethyl-heptadecane	$C_{21}H_{44}$	18344-37-1	0.01
C1	aldehyde	Methional	C_4H_8OS	3268-49-3	0.53
C2	aldehyde	Benzaldehyde	C_7H_6O	100-52-7	0.05
C3	aldehyde	Dodecanal	$C_{12}H_{24}O$	112-54-9	0.01
C4	aldehyde	Nonanal	$C_9H_{18}O$	124-19-6	0.02
D	Phenol	Ethyl maltol	$C_7H_8O_3$	4940-11-8	9.02
E1	Ketones	5-hexyldihydro-2(3H)-Furanone	$C_{10}H_{18}O_2$	706-14-9	3.48
E2	Ketones	2,5-Dimethylfuran-3,4(2H,5H)-dione	$C_6H_8O_3$	68755-49-7	0.16
E3	Ketones	1-(Methylthio)-3-pentanone	$C_6H_{12}OS$	66735-69-1	0.01
E4	Ketones	2-methyl-3-(methylthio)-Pyrazine	$C_6H_8N_2S$	2882-20-4	3.80
F1	Pyrazine	Acetylpyrazine	$C_6H_6N_2O$	22047-25-2	2.68
F2	Pyrazine	Trimethyl-pyrazine	$C_7H_{10}N_2$	14667-55-1	0.15
F3	Pyrazine	2-methyl-6-(methylthio)-pyrazine	$C_6H_8N_2S$	2884-13-1	0.62
F4	Pyrazine	2,3-dimethyl-pyrazine	$C_6H_8N_2$	5910-89-4	0.53
G1	Sour	Hexyl ester decanoic acid	$C_{16}H_{32}O_2$	10448-26-7	0.23
G2	Sour	2-hydroxy-, ethyl ester propanoic acid	$C_5H_{10}O_3$	97-64-3	0.10
G3	Sour	Pentyl ester butanoic acid	$C_9H_{18}O_2$	540-18-1	0.02
G4	Sour	n-Hexadecanoic acid	$C_{16}H_{32}O_2$	57-10-3	0.06
H	Ester	Butyl 4-methyloctanoate	$C_{13}H_{26}O_2$	NA	0.04
I	Alkene	D-Limonene	$C_{10}H_{16}$	5989-27-5	0.12
J1	Other	5-Acetyl-2,4-dimethylthiazole	C_7H_9NOS	38205-60-6	0.12
J2	Other	4-methyl-2-phenyl-1,3-dioxolane	$C_{10}H_{12}O_2$	2568-25-4	0.10
J3	Other	O-Benzoylbenzohydroximidoyl chloride	$C_{14}H_{10}ClNO_2$	NA	0.07
J4	Other	Estragole	$C_{10}H_{12}O$	140-67-0	0.01
J5	Other	3,6,9,12-Tetraoxatetradecan-1-ol	$C_{10}H_{22}O_5$	5650-20-4	0.04
